# Effectiveness and Safety of Ibrutinib for Chronic Lymphocytic Leukemia in Routine Clinical Practice: 3-Year Follow-up of the Belgian Ibrutinib Real-World Data (BiRD) Study

**DOI:** 10.1007/s44228-022-00020-8

**Published:** 2022-10-13

**Authors:** Ann Janssens, Zwi N. Berneman, Fritz Offner, Sylvia Snauwaert, Philippe Mineur, Gaetan Vanstraelen, Stef Meers, Isabelle Spoormans, Dominique Bron, Isabelle Vande Broek, Charlotte Van Bogaert, Birgit De Beleyr, Ann Smet, Lasse Nielsen, Robert Wapenaar, Marc André

**Affiliations:** 1 Department of Hematology, Universitair Ziekenhuis Leuven, Louvain, Belgium; 2 Department of Hematology, Universitair Ziekenhuis Antwerpen, Edegem, Belgium; 3 Department of Internal Medicine and Pediatrics, Ghent University Hospital, Ghent, Belgium; 4 Department of Hematology, AZ Sint-Jan Brugge, Brugge, Belgium; 5 Department of Hematology, Grand Hôpital De Charleroi - Notre Dame, Charleroi, Belgium; 6 Department of Hematology, CHR Verviers, Verviers, Belgium; 7 Department of Hematology, AZ KLINA, Antwerp, Belgium; 8 Department of Hematology, AZ Damiaan, Ostend, Belgium; 9 Department of Hematology, Institut Jules Bordet (ULB), Brussels, Belgium; 10 Department of Oncology, AZ Nikolaas, Sint Niklaas, Belgium; 11 Janssen-Cilag NV, Beerse, Belgium; 12 Janssen-Cilag BV, Breda, The Netherlands; 13 Department of Hematology, Université Catholique de Louvain, CHU UCL, Namur, Belgium

**Keywords:** Ibrutinib, Chronic lymphocytic leukemia, Belgium, Real-world evidence, Effectiveness, Safety

## Abstract

The multicenter observational BiRD study investigated the real-world effectiveness and safety of ibrutinib in patients with chronic lymphocytic leukemia (CLL), mantle cell lymphoma (MCL) and Waldenström’s macroglobulinemia (WM) in Belgium. This interim analysis reports results for patients with CLL, with a median follow-up of 34 months. Overall, patients had predominantly relapsed/refractory disease (73%) and were elderly (median age 72 years) with high-risk features such as del17p and/or *TP53* mutations (59%). Patients were included either prospectively or retrospectively, and the total patient population effectiveness results were adjusted with left truncation. In the effectiveness population (*N* = 221: prospective, *n* = 71; retrospective, *n* = 150), the overall response rate was 90.0%. Median progression-free survival was 38.3 months (prospective, not estimable; retrospective, 51.5 months) and median overall survival was not yet estimable in the total, prospective and retrospective groups. Treatment-emergent adverse events (TEAEs) for the prospective and retrospective groups are reported separately. Any-grade TEAEs of interest in the prospective/retrospective groups included infections (67.1%/60.1%), diarrhea (20.5%/10.5%), hypertension (16.4%/9.8%) and atrial fibrillation (12.3%/7.2%). Major bleeding was reported in 5.5%/3.3% of prospective/retrospective patients, with little difference observed between those receiving versus not receiving antithrombotic treatment. Discontinuations due to toxicity were reported in 10.5% of patients. Results from this interim analysis show treatment with ibrutinib to be effective and tolerable, with no new safety signals observed. Future analyses will report on longer-term follow-up.

## Introduction

Ibrutinib is a first-in-class, once-daily oral inhibitor of Bruton’s tyrosine kinase (BTK) approved in Europe as monotherapy for the treatment of adult patients with previously untreated and relapsed/refractory (R/R) chronic lymphocytic leukemia (CLL) [1].

Ibrutinib has demonstrated efficacy in treating patients with CLL in a number of phase 3 trials. Ibrutinib monotherapy showed improved progression-free survival (PFS) and overall survival (OS) versus chlorambucil in previously untreated patients with CLL aged ≥ 65 years (RESONATE-2™) [2], and also versus ofatumumab in patients with R/R CLL (RESONATE™) [3]. When used as a single agent or in combination with rituximab, ibrutinib therapy showed improved PFS compared with bendamustine plus rituximab in previously untreated patients with CLL aged ≥ 65 years (Alliance) [4]. It also improved PFS and OS as part of combination therapy with rituximab versus fludarabine, cyclophosphamide and rituximab (FCR) in previously untreated patients aged ≤ 70 years (E1912) [5]. Improved PFS was also shown for ibrutinib in combination with obinutuzumab versus chlorambucil plus obinutuzumab in previously untreated CLL (iLLUMINATE) [6] and in combination with bendamustine and rituximab (BR) versus placebo plus BR in patients with R/R CLL (HELIOS) [7, 8].

The Belgian ibrutinib Real-World Data study (BiRD) was designed to investigate the effectiveness and safety of ibrutinib treatment in real-world clinical practice in patients with CLL, MCL or Waldenström’s macroglobulinemia (WM) in Belgium. Results from first and second interim analyses from BiRD have been presented previously in patients with CLL and MCL [9–11], and some of the third interim analysis results in patients with CLL and MCL have also been presented [12]. Here we report the results for the cohort of patients with CLL from the third interim analysis of the BiRD study.

## Patients and Methods

### Study Design

BiRD is a retrospective and prospective, multicenter, non-interventional observational study of adult patients with a confirmed diagnosis of CLL, MCL or WM from 26 sites in Belgium who initiated reimbursed ibrutinib therapy on or after its commercial availability in Belgium (August 1, 2015 for R/R CLL and MCL, September 1, 2016 for WM and May 1, 2017 for first-line CLL), or who participated in the Medical Need Program for CLL, WM or MCL, or received free of charge ibrutinib as a single-patient request for WM and switched to reimbursed ibrutinib. Patients who initiated reimbursed ibrutinib therapy prior to the inclusion visit were eligible for retrospective enrollment in the study, regardless of whether the ibrutinib therapy was ongoing at the time of inclusion (retrospective inclusion). Patients who had begun ibrutinib therapy at the time of inclusion in the study were added prospectively. This is the third of four planned interim analyses. The first occurred after the last patient was enrolled, the second after 18 months, this present and third at 3 years and the fourth is planned for early 2023. However, ad hoc interim analyses may also be conducted by the sponsor, based on specific questions from the medical field or from health authorities. Only patients with CLL are included in this analysis, and the cutoff date was March 31, 2020.

Each patient was treated according to the standard of care; their decision to take part in the observational study did not influence their medical care. Therapy decisions were made at the discretion of the participating physician, according to routine clinical practice.

Data were collected every 3 months during the first year and every 6 months thereafter for a period of 5 years. Data were collected for ibrutinib treatment until discontinuation, after which the follow-up period for response started with any subsequent therapy. If patients received no further treatment, they were followed up for survival only. Safety data were collected for some patients who began ibrutinib before inclusion and yet continued with ibrutinib therapy; therefore, there was a retrospective/prospective safety data collection period for these patients—defined within the retrospective group as “before inclusion” and “after inclusion.”

The study protocol was approved by the institutional review boards or independent ethics committees of participating centers. All patients received oral and written information on the study and provided informed consent to data collection and source data verification.

### Inclusion and Exclusion Criteria

Patients had to be aged ≥ 18 years, with a confirmed diagnosis of CLL, and eligible for reimbursed ibrutinib treatment, according to the National Institute for Health and Disability Insurance (RIZIV/INAMI). This was both before and after the commercial availability of ibrutinib and, therefore, also included patients participating in a Medical Need Program who were switched to reimbursed ibrutinib treatment when it became available (August 1, 2015). Previously untreated patients without del17p or *TP53* mutation who were fit to receive fludarabine-based regimens were not eligible for ibrutinib treatment according to Belgian treatment guidelines at the start of the study. Reimbursement criteria for CLL are based on the International Workshop on Chronic Lymphocytic Leukemia (iwCLL) criteria [13] and include del17p or *TP53* mutation or R/R disease. Patients were excluded if they were participating in another investigational or clinical study or in any expanded access program during the ibrutinib treatment period covered by this observational study.

### Outcome Measures

The primary outcome measures were investigator-assessed overall response rate (ORR) and PFS. Secondary outcome measures included OS and safety, which includes treatment exposure and treatment-emergent adverse events (TEAEs).

PFS was defined as the time from the start of ibrutinib treatment to progression or death from any cause. OS was defined as the time in months from initiation of ibrutinib to death. ORR was the sum of complete response (CR), partial response (PR) and partial response with lymphocytosis, as assessed by the investigator. Although confirmation of CR by minimal residual disease analysis of bone marrow was possible, it is not routinely performed in real-world practice, and this definition of CR should be considered unconfirmed in most cases. After discontinuation of ibrutinib therapy, during the treatment-free period, patients were followed for response or disease progression every 6 months ± 2 weeks. If no visit was conducted within this recommended period, data from the visit conducted as close as possible to the protocol-specified time point were recorded.

All TEAEs were collected prospectively, whereas only adverse drug reactions considered related to ibrutinib were collected retrospectively.

### Statistics

This study describes everyday ibrutinib treatment practice for CLL. The effectiveness population included all patients who met the inclusion criteria and who received at least one dose of ibrutinib and the safety population included all patients who received at least one dose of ibrutinib.

All time-to-event variables were analyzed using standard survival analysis methods, including Kaplan–Meier product-limit survival curve. The median time to event with two-sided 95% confidence intervals (CIs) was estimated. All continuous variables were summarized using descriptive statistics, which included the number of patients, mean, standard deviation, median, interquartile range (IQR) minimum and maximum and 95% CI. Data were reported for the prospective, retrospective and/or total patient populations for the effectiveness population. Owing to the differences in adverse event collection between the retrospective and prospective groups, these data are presented separately and are all referred to as “TEAEs” for ease of notation. The number and percentage of patients who experienced at least one occurrence of each TEAE (presented as preferred term and categorized by system organ class) were summarized. TEAEs were not graded but were summarized by severity for serious and severe TEAEs, according to the worst experienced. Patients who died before enrollment in the trial could not be included according to Belgian law, as no informed consent could be obtained. For this reason, the total population effectiveness results were adjusted with left truncation, which reduces part of the follow-up time of the retrospective patients to avoid any mortality bias. However, this shortened the median time to event and, as the retrospective group is larger than the prospective group, this also affected the median time to event of the combined analysis.

## Results

### Patient Demographics and Baseline Characteristics

A total of 221 evaluable patients with CLL were included in this interim analysis for effectiveness (retrospective, *n* = 150; prospective, *n* = 71). More than 80% of patients initiated reimbursed ibrutinib, with the remainder participating in the Medical Need Program. The median age at ibrutinib initiation was 72 (range 38–90) years, 64.3% of patients were male, 86.6% had an Eastern Cooperative Oncology Group performance status of 0–1, and 17.8% had a history of significant cardiovascular (CV) disease. Ongoing atrial fibrillation (AF) and other ongoing CV disease were both reported by 14/38 patients. Table 1 details the patient characteristics at baseline.

**Table 1:** Patient baseline characteristics for CLL population

	Prospective *n* = 71	Retrospective *n* = 150	Overall *N* = 221
Age at ibrutinib initiation, median (range), years	71 (50–88)	72 (38–90)	72 (38–90)
Male, *n* (%)	51 (71.8)	91 (60.7)	142 (64.3)
Time from diagnosis to ibrutinib initiation, median (range), years	4.7 (0–24.1)	6.1 (0–31.4)	5.8 (0–31.4)
Number of prior lines of therapy, *n* (%)	*n* = 70	*n* = 149	*N* = 219
0	30 (42.9)	30 (20.1)	60 (27.4)
1	15 (21.4)	38 (25.5)	53 (24.2)
2	18 (25.7)	43 (28.9)	61 (27.9)
≥ 3	7 (10.0)	38 (25.5)	45 (20.5)
Patients with prior stem cell transplant, *n* (%)	*n* = 69 1 (1.4)	*n* = 148 5 (3.4)	*N* = 217 6 (2.8)
ECOG PS, *n* (%)	*n* = 53	*n* = 111	*N* = 164
0	21 (39.6)	57 (51.4)	78 (47.6)
1	23 (43.4)	41 (36.9)	64 (39.0)
2	7 (13.2)	10 (9.0)	17 (10.4)
3	1 (1.9)	3 (2.7)	4 (2.4)
4	1 (1.9)	0 (0.0)	1 (0.6)
del17p present and/or *TP53* mutated, *n* (%)	*n* = 43 31 (72.1)	*n* = 89 47 (52.8)	*N* = 132 78 (59.1)
del11q present, *n* (%)	*n* = 33 5 (15.2)	*n* = 93 28 (30.1)	*N* = 126 33 (26.2)
IGHV unmutated, *n* (%)	*n* = 27 17 (63.0)	*n* = 53 41 (77.4)	*N* = 80 58 (72.5)
Complex karyotype, *n* (%)	*n* = 34 17 (50.0)	*n* = 71 44 (62.0)	*N* = 105 61 (58.1)
History of significant CV disease^a^	*n* = *70* 14 (20.0)	*n* = *143* 24 (16.8*)*	*n* = *213* 38 (17.8)
Ongoing AF^a^	*n* = *14* 7 (50.0)	*n* = *24* 7 (29.2)	*n* = *38* 14 (36.8)
Other ongoing CV disease^a^	*n* = *14* 6 (42.9)	*n* = *24* 8 (33.3)	*n* = *38* 14 (36.8)

*AF* atrial fibrillation, *CLL* chronic lymphocytic leukemia, *CV* cardiovascular, *ECOG PS* Eastern Cooperative Oncology Group performance status

^a^As assessed by the investigator

The median time from diagnosis to ibrutinib initiation was 5.8 years and 72.6% patients had at least one prior line of therapy. In total, 78 of 132 assessed patients (59.1%) had del17p and/or *TP53* mutation, and 72.5% of only 80 patients assessed had unmutated IGHV. In line with reimbursement criteria, 43.6% of patients with del17p and/or *TP53* mutations had no prior lines of therapy, whereas 71.7% of patients without del17p and *TP53* mutations had at least one prior line of therapy.

The majority of patients (88.1%) initiated ibrutinib therapy at the recommended dose of 420 mg daily.

### Effectiveness

**Fig. 1:**
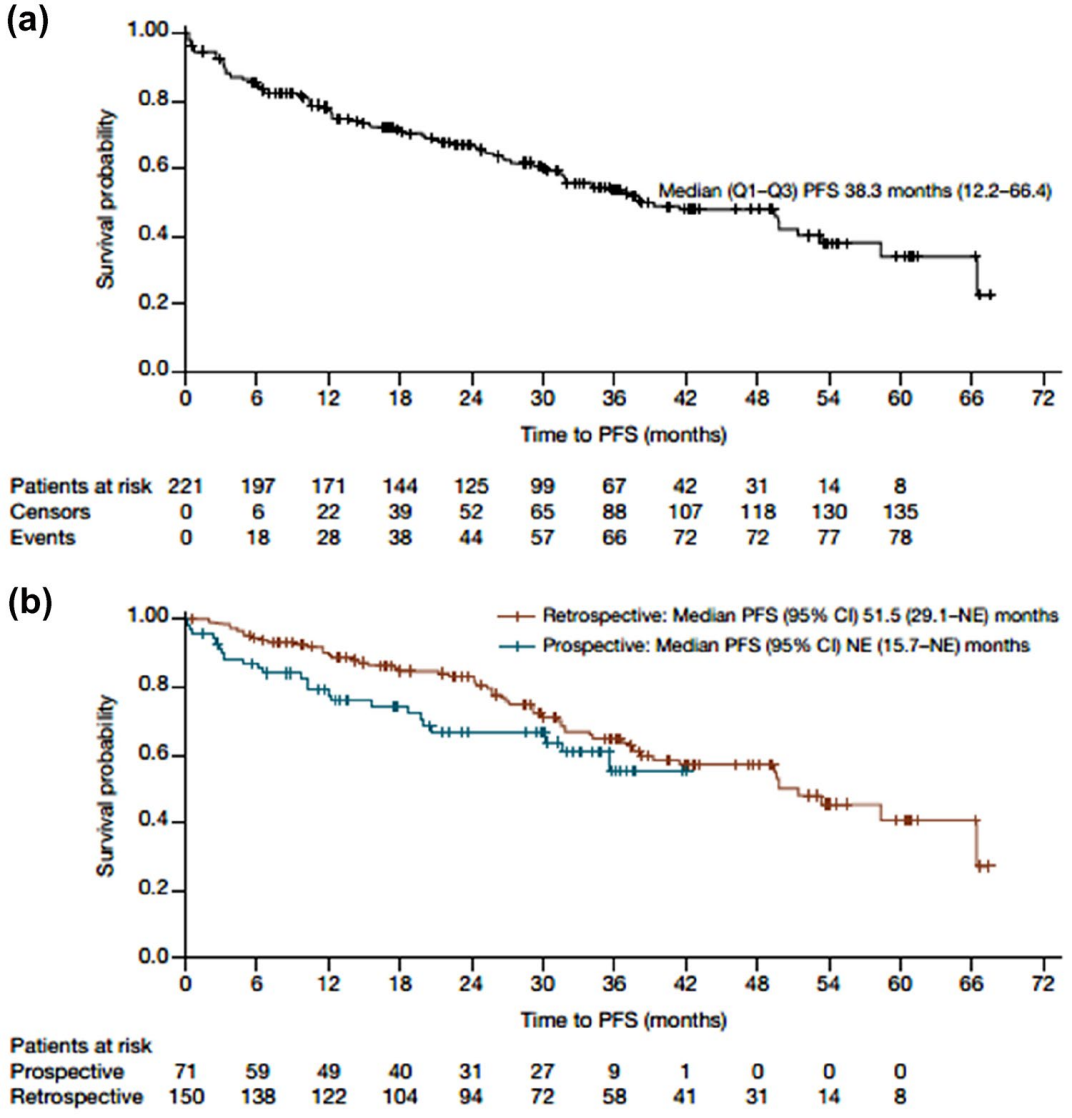
**a** PFS for total population corrected with left truncation. **b** PFS for prospective and retrospective populations

**Fig. 2:**
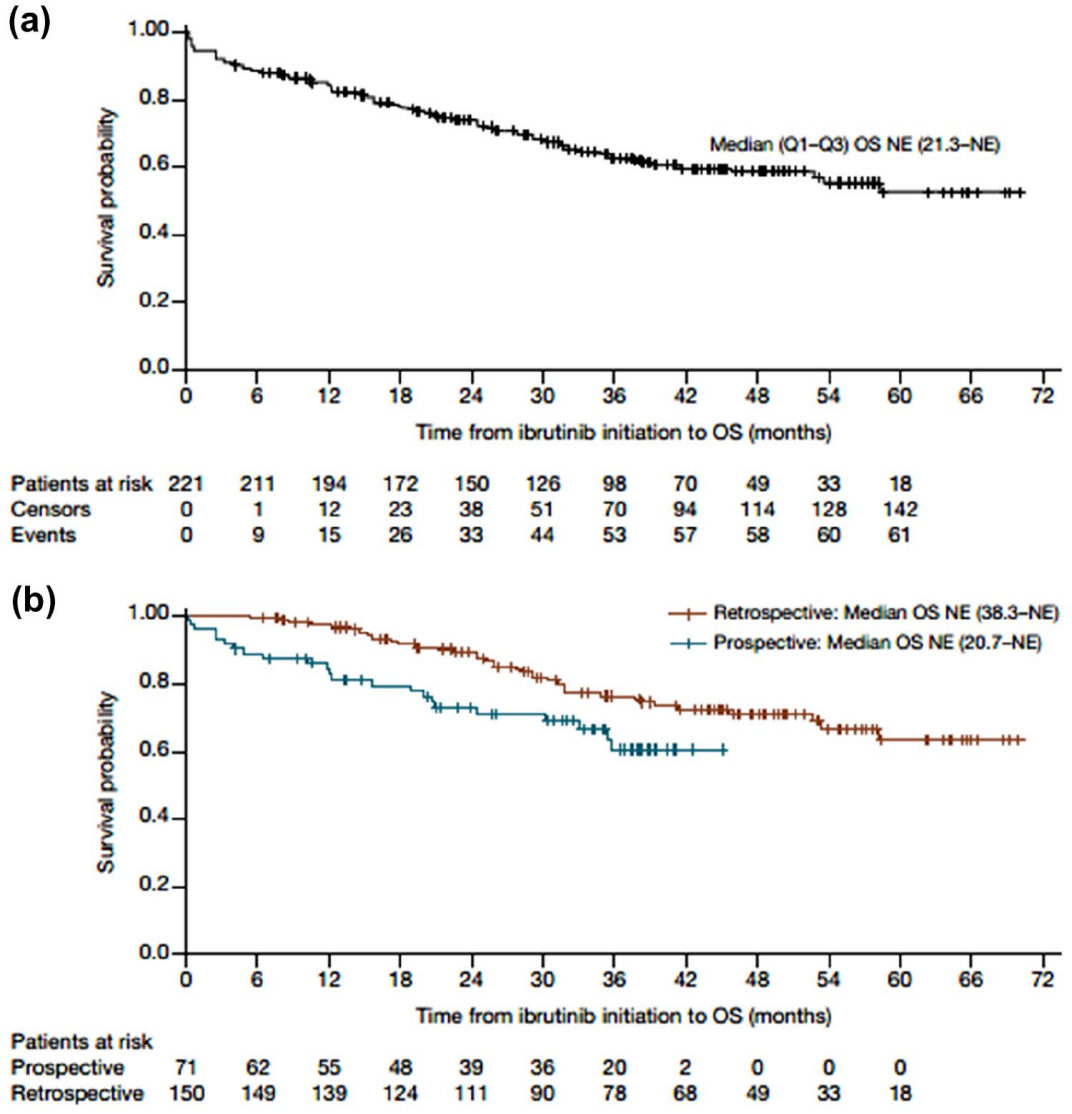
**a** OS for total population corrected with left truncation. **b** OS for prospective and retrospective populations

The median (range) follow-up was 34.3 (0.2–69.9) months in the total population. Table 2 details the main effectiveness outcomes. The cumulative ORR (best response at 60 months) was 90.0% (CR 16.7%; PR 51.6%; PR with lymphocytosis, 21.7%). The median (Q1–Q3) PFS for CLL was 38.3 (12.2–66.4) months for the total population, not estimable (NE) (15.7–NE) in the prospective group and 51.5 (29.1–NE) months in the retrospective group (Fig. 1). PFS was longer in patients without del17p and *TP53* mutations than in those with del17p and/or *TP53* mutations (49.9 versus 41.5 months; Table 2). There was little difference in PFS in patients with mutated or unmutated IGHV (49.9 versus 53.5 months). PFS varied slightly by line of therapy, but the variation did not follow any particular pattern. PFS was longer in patients younger than 65 years compared with those older than 65 years (49.5 versus 36.8 months). Median (Q1–Q3) OS was not estimable (21.3–NE) in the total population and was also not estimable in the prospective and retrospective groups (Fig. 2). The 36-month OS rate was 62.9% overall and 60.6% and 76.3% in the prospective and retrospective groups, respectively. Median time to next treatment was 35.7 months overall and not estimable and 51.5 months in the prospective and retrospective groups, respectively. The numbers obtained for the total population are a result of the statistical analysis used, as detailed in the methods.

**Table 2:** Response outcomes

	Prospective	Retrospective	Total^a^
*N*	71	150	221
Median PFS, months (Q1–Q3)	NE (15.7–NE)	51.5 (29.1–NE)	38.3 (12.2–66.4)
PFS: del17p and/or *TP53* mutation, months (Q1–Q3)	NE^b^	49.7 (27.3–NE) *n* = 47	41.5 (21.3–NE) *n* = 78
PFS: No del17p and *TP53* mutation, months (Q1–Q3)	NE^b^	49.9 (31.3–NE) *n* = 42	49.9 (17.8–NE) *n* = 54
PFS: Mutated IGHV, months (Q1–Q3)	NE^b^	49.9 (37.3–49.9) *n* = 12	49.9 (21.3–49.9) *n* = 22
PFS: Unmutated IGHV, months (Q1–Q3)	NE^b^	53.5 (31.6–NE) *n* = 41	53.5 (31.6–NE) *n* = 58
PFS: No prior LOTs, months (Q1–Q3)	NE (35.8–NE) *n* = 30	NE (26.6–NE) *n* = 30	35.7 (26.6–NE) *n* = 60
PFS: 1 prior LOT, months (Q1–Q3)	NE^c^ (10.3–NE) *n* = 40	49.9 (29.1–NE) *n* = 38	31.7 (9.4–NE) *n* = 53
PFS: 2 prior LOTs, months (Q1–Q3)	–^c^	58.4 (25.7–NE) *n* = 43	36.8 (10.3–66.4) *n* = 61
PFS: ≥ 3 prior LOTs, months (Q1–Q3)	–^c^	51.5 (31.6–NE) *n* = 38	37.3 (12.2–NE) *n* = 45
PFS: age ≤ 65 years, months (Q1–Q3)	NE (20.0–NE) *n* = 19	58.4 (34.3–66.4) *n* = 40	49.5 (13.9–66.4) *n* = 59
PFS: age > 65 years, months (Q1–Q3)	NE (12.2–NE) *n* = 52	49.9 (27.3–NE) *n* = 110	36.8 (12.2–NE) *n* = 162
36-month PFS rate, % (95% CI)	55.6 (38.5–69.7)	65.0 (55.5–73.0)	53.4 (–)^d^
Time to best response, months (Q1–Q3)	4.1 (2.3–8.7)	6.4 (3.0–12.7)	4.4 (2.3–9.7)
Duration of response, months (Q1–Q3)	NE (NE–NE)	63.7 (37.5–NE)	63.7 (31.5–NE)
Median OS, months (Q1–Q3)	NE (20.7–NE)	NE (38.3–NE)	NE (21.3–NE)
36-month OS rate, % (95% CI)	60.6 (45.8–72.6)	76.3 (67.8–82.9)	62.9 (–)^d^
Time to next treatment, months (Q1–Q3)	NE (12.5–NE)	51.5 (24.4–NE)	35.7 (11.7–NE)
Time to progression, months (Q1–Q3)	NE (NE–NE)	66.4 (37.3–NE)	66.4 (34.0–NE)
Time to permanent discontinuation, months (Q1–Q3)	NE (15.8–NE)	51.5 (16.7–NE)	41.2 (10.8–NE)

*CI* confidence interval, *LOT* line of therapy, *NE* not estimable, *OS* overall survival, *PFS* progression-free survival

^a^Analysis corrected with left truncation

^b^Patient numbers too small to give meaningful results, so analysis not performed

^c^Value is combined for ≥ 1 prior line of therapy

^d^95% CI not calculated as analysis corrected with left truncation

### Safety

There were 226 patients in the safety analysis population, 73 in the prospective group and 153 in the retrospective group. The median (range) duration of ibrutinib treatment in the total safety population was 25.5 (0.1–70.0) months; it was longer in the retrospective (28.6 [0.4–70.0]), than in the prospective group (19.6 [0.1–45.1]) months. At treatment initiation, most patients (88.1%) were prescribed a daily dose of 420 mg ibrutinib and this was comparable in the prospective and retrospective groups (88.9% and 86.3%, respectively).

A total of 123 (54.4% of 226) patients discontinued treatment during the analysis period, 38 in the prospective and 85 in the retrospective group. The overall reasons for discontinuation, when given, were disease progression (*n* = 29; 12.8%), toxicity (*n* = 26; 11.5%), death (*n* = 21; 9.3%), other (*n* = 18; 8.0%), comorbidities (*n* = 6; 2.7%), physician preference (*n* = 5; 2.2%) and one each (0.4%) of concomitant medication, patient preference and surgery. None of the deaths were reported as sudden cardiac deaths, and the one cardiac-related death (cardiac arrest) reported was considered not related to treatment by the investigator. Only two deaths were considered very likely related to ibrutinib by the investigators, pneumonia and aspergillus infection.

As TEAEs consisting of all events related and unrelated to treatment that occurred during the study were reported for the prospective group, and only adverse events considered related to ibrutinib were reported for the retrospective group, they are reported separately for the two patient groups. TEAEs of interest are detailed in Table 3. For the prospective and retrospective groups, respectively, 100% and 83.7% of patients had at least one TEAE, while 58.9% and 43.8% of patients had at least one serious TEAE. In the prospective and retrospective groups, respectively, TEAEs led to ibrutinib dose reduction in 21.9% and 10.5% of patients, dose interruption in 39.7% and 23.5%, and withdrawal (which includes any discontinuations related or unrelated to ibrutinib therapy, including death, toxicity, comorbidities, progressive disease and physician preference) in 26.0% and 19.6%.

**Table 3:** Treatment-emergent adverse events (TEAE) of interest

*n* (%)	Retrospective *n* = 153		Prospective *n* = 73
	Before inclusion^a^	After inclusion	
Patients with TEAEs (any grade)			
≥ 1 TEAE	–	128 (83.7)	73 (100)
≥ 1 TEAE related to ibrutinib	114 (74.5)	99 (64.7)	62 (84.9)
≥ 1 severe TEAE^b^	–	61 (39.9)	44 (60.3)
≥ 1 serious TEAE^c^	–	67 (43.8)	43 (58.9)
≥ 1 serious TEAE related to ibrutinib	16 (10.5)	36 (23.5)	15 (20.5)
≥ 1 TEAE leading to drug withdrawal^d^	–	30 (19.6)	19 (26.0)
≥ 1 TEAE leading to death	–	19 (12.4)	14 (19.2)
Patients with treatment-emergent bleeding events			
≥ 1 bleeding event	–	46 (30.1)	36 (49.3)
≥ 1 major bleeding event^c^	–	5 (3.3)	4 (5.5)
≥ 1 bleeding event while on antithrombotic treatment^d^	–	18 (42.9)	22 (78.6)
≥ 1 bleeding event while not on antithrombotic treatment^e^		28 (25.2)	14 (31.1)
≥ 1 major bleeding event while on antithrombotic treatment^e,f^	–	0	3 (10.7)
≥ 1 major bleeding event while not on antithrombotic treatment^e,f^		4 (3.6)	0
Patients with ≥ 1 TEAE of interest (any grade)			
Infection^g^	–	92 (60.1)	49 (67.1)
Hypertension	–	15 (9.8)	12 (16.4)
Arrhythmia^g^	–	15 (9.8)	16 (21.9)
Atrial fibrillation	–	11 (7.2)	9 (12.3)
Arrhythmia	–	0	4 (5.5)
Arthralgia/Myalgia^g^	–	12 (7.8)	18 (24.7)
Arthralgia	–	7 (4.6)	15 (20.5)
Myalgia	–	5 (3.3)	7 (9.6)
Diarrhea	–	16 (10.5)	15 (20.5)
Rash^g^	–	12 (7.8)	11 (15.1)

*TEAE* treatment-emergent adverse event

^a^For retrospectively included patients, only reported ibrutinib-related adverse events were recorded from the period prior to inclusion in the study

^b^Severe TEAE: on a scale of mild/moderate/severe and usually requires medical assistance/intervention/therapy and may require hospitalization

^c^Serious TEAE: life threatening or causing death

^d^Withdrawal includes any discontinuations that are related or unrelated to ibrutinib therapy, including death, toxicity, comorbidities, progressive disease and physician preference

^e^Major bleeding is a severe/serious bleeding event

^f^Percentages are calculated based on the numbers of patients with and without concomitant antithrombotic therapy in the retrospective group (42 and 111, respectively) and in the prospective group (28 and 45, respectively)

^g^Grouped terms

Some patients were taking concomitant antithrombotic therapy during the study, 28 in the prospective and 42 in the retrospective group. Bleeding was more frequent in patients on such therapy compared with those not, 78.6% versus 31.1% in the prospective and 42.9% versus 25.2% in the retrospective group, and major bleeding was infrequent, 5.5% in the prospective group and 3.3% in the retrospective group. The most common antithrombotic therapy was an antiplatelet agent, 46.4% in the prospective group and 45.2% in the retrospective group, followed by a non-vitamin K oral anticoagulant, 10.7% in the prospective group and 21.4% in the retrospective group, vitamin K antagonist, none in the prospective group and 4.8% in the retrospective group, or other therapy, 14.2% in the prospective group and 9.5% in the retrospective group. More than one antithrombotic treatment was used by 28.6% of patients taking antithrombotic therapy in the prospective group and by 19.0% in the retrospective group; this included patients who switched therapies and, therefore, these patients were not all taking several antithrombotic agents concomitantly.

## Discussion

BiRD is an ongoing real-world study that aims to evaluate the effectiveness and safety of ibrutinib in patients with CLL in Belgium. Those enrolled in BiRD represent the clinical spectrum of a real-world CLL population, as they were mostly elderly (median age 72 years), and approximately three quarters had been treated previously. Almost half of patients with del17p and/or *TP53* mutations had no prior lines of therapy, yet nearly three quarters of patients without del17p and *TP53* mutations had at least one prior line of therapy, which is driven by the indication and reimbursement criteria in Belgium. It is encouraging that the vast majority of patients (88%) in this real-world population in normal clinical practice received the recommended dose of ibrutinib at treatment initiation. As many patients were included during the early years of ibrutinib market authorization, it is possible that physicians were initially cautious with dosing, starting at a dose lower than recommended, and titrating up to ensure tolerability.

In this third interim analysis, the median follow-up for effectiveness was 34.3 months although there was a wide range, up to 70 months. The cumulative ORR up to 60 months was 90.0%, a finding similar to the ORR reported in the second interim analysis at 12 months (86.9%) [9], and also similar to some clinical trials with high-risk populations. In the RESONATE™ trial of single-agent ibrutinib in patients with R/R CLL, which had a comparable percentage of high-risk patients (32% with del17p, 51% with *TP53* mutation; 86% considered high risk in the ibrutinib arm), the cumulative ORR for ibrutinib at the 6-year follow-up was 91% [14], and in a phase 2 study with high-risk patients (63% with del17p and/or *TP53* mutation), the ORR for ibrutinib was 96% at a follow-up of 6 months [15]. High rates of response have also been noted with ibrutinib treatment in clinical trials without such a high-risk population, such as the RESONATE-2™ study in previously untreated patients without del17p over the age of 65, which reported an ORR of 92% with up to 66 months follow-up [16]. Other real-world studies have also produced similar levels of response to ibrutinib, including the FIRE real-world study, which is a similar design to our BiRD study, yet conducted in France. FIRE reported an ORR of 89.6% in the overall CLL patient group at a follow-up of 21.6 months in a population of patients with 58.7% del17p and/or *TP53* mutations [17]. In addition, a Danish retrospective cohort study in 205 patients, 72.1% of whom had del17p or *TP53* mutations, with a median follow-up of 21.4 months, reported an ORR of 76.4% [18], and an analysis of 95 Swedish patients, 62% with del17p or *TP53* mutations, treated with ibrutinib in a compassionate use program reported an ORR of 84% [19].

In our study, the median PFS was 38.3 months for the total population, which is unchanged from the second interim analysis at a median follow-up of 20.9 months [9]. Interestingly, the PFS values for the separate prospective and retrospective groups were both longer than this value (NE and 51.5 months, respectively), which is a result of the statistical analysis on the total cohort. As those patients in the retrospective group who died prior to study entry could not be included because they could not provide consent, an adjustment was made with left truncation to avoid bias in the retrospectively included patients on the total population analysis. As the retrospective group is larger than the prospective group of patients, this impacts the results for the total CLL cohort. The PFS values reported in our analysis are all within the range of those reported in clinical trials, such as RESONATE™, with a PFS of 44.1 months at 6 years’ follow-up [14], and RESONATE-2™, with a PFS not estimable after a median follow-up of 60 months [16]. In the FIRE real-world study of similar design, the overall PFS at median follow-up of 21.6 months was 37.7 months, and was not estimable in either the prospective or retrospective patient groups at 15.2 and 29.6 months’ median follow-up, respectively [17]. In the Danish real-world study, the median PFS was 41.2 months [18].

PFS was shorter in patients with del17p and *TP53* mutations (42 months compared with 50 months), yet this compares favorably with the PFS of 11 months reported for FCR treatment in previously untreated patients with *TP53* alterations after 5 years’ follow-up [20]. Poor responses to chemotherapy in these high-risk patients have been reported previously [21], yet ibrutinib therapy is associated with durable responses [15, 22]. In patients with mutated or unmutated IGHV, the PFS was similar, as reported previously for ibrutinib [16]; however, only 60% of patients in our study underwent cytogenetic testing for del17p or *TP53* and even fewer (36%) were tested for IGHV (it must be noted, though, that IGHV testing was not reimbursed for patients over 65 years of age in Belgium at the start of the study, and is currently only reimbursed for patients eligible for chemoimmunotherapy treatment). This highlights a potentially important gap in testing, a finding also noted in another real-world study in the United States [23], which could increase the likelihood of patients not receiving optimal treatment in clinical practice. Cytogenetic testing is now becoming more common, and this will help to improve treatment choices.

As expected, younger patients (aged < 65 years) had longer PFS than those aged ≥ 65 years, and median PFS varied slightly by number of previous lines of therapy. This variation did not follow any particular pattern, partly due to low patient numbers in each group and the long PFS values at this time of follow-up. Interestingly, this non-correlation of PFS with prior therapy line was also noted in a retrospective US real-world cohort analysis [24]. Patients were enrolled into our study at the beginning of ibrutinib use, when practice policies were adapted, and it is therefore likely that early recruits were of higher risk than later ones, and that better results may be expected with longer follow-up, particularly in previously untreated patients.

The median OS in our study was not estimable, as also reported in several other studies at time points shorter than several years [7, 8, 16, 17]. The estimated 36-month OS rate of 62.9% is in the expected range, when considering the OS rate of 63% at 30 months in the Swedish cohort study [19] and in other real-world studies reporting at earlier time points: the Danish cohort study showed an estimated OS of 76.8% at 24 months [18], and the FIRE study estimated a value of 86.9% at the same 24-month time point [17]. It is likely that further follow-up is needed to determine additional information on survival.

In the safety analysis with up to 70 months of follow-up, fewer TEAEs were reported in the retrospective than in the prospective group, as only events considered related to ibrutinib were reported retrospectively. In general, TEAEs were in the range of those previously reported in real-world studies, including FIRE [17], the Denmark cohort [18] and the Swedish cohort [19], and within the range of single-agent ibrutinib clinical trials of CLL [2, 3, 14]. Patients treated in a real-world setting, however, may be expected to have higher rates of TEAEs and treatment discontinuation than those in a clinical trial setting, because a real-world population includes higher risk patients who might be excluded from clinical trials. The rate of discontinuations due to toxicity observed in our study (11.5%) was lower than that reported in other real-world studies (20% in the Swedish retrospective study [19], 22.9% in the Danish retrospective study [18], 20.8% in a US cohort study [24] and 17.5% in a large UK real-world study from 2016 of 315 patients after 16 months median follow-up [25]). These older studies, however, may have included higher-risk populations in the earlier days of ibrutinib use, particularly with compassionate use, potentially leading to higher discontinuations. The frequency of bleeding in our study was similar to that experienced in clinical trials and real-world studies [7, 8, 16, 17]. Bleeding was more frequent in patients receiving antithrombotic therapy, but major bleeding events were too infrequent to determine any correlation. The frequency of TEAEs varied little from the second [10] to the third interim analysis of BiRD, which is to be expected as TEAEs are reported by actual study time and not by treatment course per patient. It is reassuring that no cardiac deaths or sudden deaths were reported during this long follow-up period. Overall, treatment with ibrutinib was tolerable and no new safety signals were observed.

There are a number of limitations to consider when evaluating the results from this analysis. Unlike clinical trials, the effectiveness and safety parameters are presented through descriptive data in a real-world setting and assessed by the investigators. The response was assessed by physicians in routine clinical practice, and therefore comparison with clinical trials is challenging. The differences between the retrospective and prospective populations are evident, and adjustments to the overall population aimed to reduce bias, although bias cannot be entirely discounted.

This large, real-world study gives a unique perspective of ibrutinib use in a single country, encompassing most major hematology centers in Belgium. It provides information on the long-term use of ibrutinib in CLL in real-world clinical practice, with up to 7 years (median 34 months) follow-up. It is promising to see that results from randomized trials in more restricted populations have translated into real world with ibrutinib.

## Conclusions

Results from this large, real-world study show ibrutinib to be effective in this mostly elderly CLL population, with results in alignment with those from randomized controlled trials and with other real-world studies. TEAEs were in line with those from other studies, few patients discontinued due to toxicity and no new safety signals were evident. We await further follow-up from the BiRD study to confirm the longer-term effectiveness and safety of ibrutinib.

## Data Availability

The data-sharing policy of Janssen Pharmaceutical Companies of Johnson & Johnson is available at https://www.janssen.com/clinical-trials/transparency. As noted on this site, requests for access to the study data can be submitted through Yale Open Data Access (YODA) Project site at http://yoda.yale.edu.

## Abbreviations

AF: Atrial fibrillation
BiRD: Belgian Ibrutinib Real-World Data
BR: Bendamustine and rituximab
BTK: Bruton’s tyrosine kinase
CI: Confidence interval
CLL: Chronic lymphocytic leukemia
CR: Complete response
CV: Cardiovascular
ECOG PS: Eastern Cooperative Oncology Group performance status
FCR: Fludarabine, cyclophosphamide and rituximab
IQR: Interquartile range
iwCLL: International Workshop on Chronic Lymphocytic Leukemia
LOT: Line of therapy
MCL: Mantle cell lymphoma
NE: Not estimable
ORR: Overall response rate
OS: Overall survival
PFS: Progression-free survival
PR: Partial response
R/R: Relapsed/refractory
TEAE: Treatment-emergent adverse event
WM: Waldenström’s macroglobulinemia
